# Obstetric hemorrhage and shock management: using the low technology Non-pneumatic Anti-Shock Garment in Nigerian and Egyptian tertiary care facilities

**DOI:** 10.1186/1471-2393-10-64

**Published:** 2010-10-18

**Authors:** Suellen Miller, Mohamed MF Fathalla, Oladosu A Ojengbede, Carol Camlin, Mohammed Mourad-Youssif, Imran O Morhason-Bello, Hadiza Galadanci, David Nsima, Elizabeth Butrick, Tarek al Hussaini, Janet Turan, Carinne Meyer, Hilarie Martin, Aminu I Mohammed

**Affiliations:** 1 Department of Obstetrics, Gynecology and Reproductive Sciences, University of California, San Francisco, San Francisco, USA; 2 Department of Obstetrics and Gynecology, Faculty of Medicine, Assiut University Women's Health Center, Assiut, Egypt; 3 Center for Population and Reproductive Health, College of Medicine/University College Hospital, University of Ibadan, Ibadan, Nigeria; 4 Department of Obstetrics and Gynecology, El Galaa Maternity Teaching Hospital, Cairo, Egypt; 5 Aminu Kano Teaching Hospital, Kano, Nigeria; 6 Department of Obstetrics and Gynecology, Katsina General Hospital, Katsina, Nigeria; 7 Murtala Mohammed Specialist Hospital, Kano, Nigeria; 8 Current Address: Saint Joseph's Medical Center, Yonkers, New York, USA

## Abstract

**Background:**

Obstetric hemorrhage is the leading cause of maternal mortality globally. The Non-pneumatic Anti-Shock Garment (NASG) is a low-technology, first-aid compression device which, when added to standard hypovolemic shock protocols, may improve outcomes for women with hypovolemic shock secondary to obstetric hemorrhage in tertiary facilities in low-resource settings.

**Methods:**

This study employed a pre-intervention/intervention design in four facilities in Nigeria and two in Egypt. Primary outcomes were measured mean and median blood loss, severe end-organ failure morbidity (renal failure, pulmonary failure, cardiac failure, or CNS dysfunctions), mortality, and emergency hysterectomy for 1442 women with ≥750 mL blood loss and at least one sign of hemodynamic instability. Comparisons of outcomes by study phase were assessed with rank sum tests, relative risks (RR), number needed to treat for benefit (NNTb), and multiple logistic regression.

**Results:**

Women in the NASG phase (n = 835) were in worse condition on study entry, 38.5% with mean arterial pressure <60 mmHg vs. 29.9% in the pre-intervention phase (p = 0.001). Despite this, negative outcomes were significantly reduced in the NASG phase: mean measured blood loss decreased from 444 mL to 240 mL (p < 0.001), maternal mortality decreased from 6.3% to 3.5% (RR 0.56, 95% CI 0.35-0.89), severe morbidities from 3.7% to 0.7% (RR 0.20, 95% CI 0.08-0.50), and emergency hysterectomy from 8.9% to 4.0% (RR 0.44, 0.23-0.86). In multiple logistic regression, there was a 55% reduced odds of mortality during the NASG phase (aOR 0.45, 0.27-0.77). The NNTb to prevent either mortality or severe morbidity was 18 (12-36).

**Conclusion:**

Adding the NASG to standard shock and hemorrhage management may significantly improve maternal outcomes from hypovolemic shock secondary to obstetric hemorrhage at tertiary care facilities in low-resource settings.

## Background

Obstetric hemorrhage is the leading cause of maternal mortality globally. In low-resource settings, delays in identifying hemorrhage, delays in transport to facilities from a home birth or from primary care centers, and delays in receiving definitive therapies upon arrival at tertiary facilities contribute to high rates of maternal mortality and morbidity secondary to hypovolemic shock [1]. Access to definitive therapies, such as blood transfusions and surgery, necessary to manage severe hypovolemia, is limited to major medical centers (tertiary facilities), which are often located in large cities. Even in advanced university teaching hospitals, there may be hours of delay awaiting blood transfusions, there may be no blood bank or no electricity for the blood bank, and/or a lack of blood donors. In the absence of hemodynamic stability provided by those transfusions, anesthetists and surgeons (if they are available) will not consider performing necessary hemostatic surgery. Further, in some settings, if the woman is eligible for surgery, it is more feasible to perform an emergency hysterectomy than the more time and skill intensive devascularization. A woman may unnecessarily lose her uterus and suffer infertility, which in certain cultures may lead to divorce and increasing poverty. In a 2003 Bellagio meeting on the problem of maternal mortality and morbidity due to hemorrhage, experts suggested trying anti-shock garments (ASG) for stabilization to help women survive delays [2].

**Figure 1:**
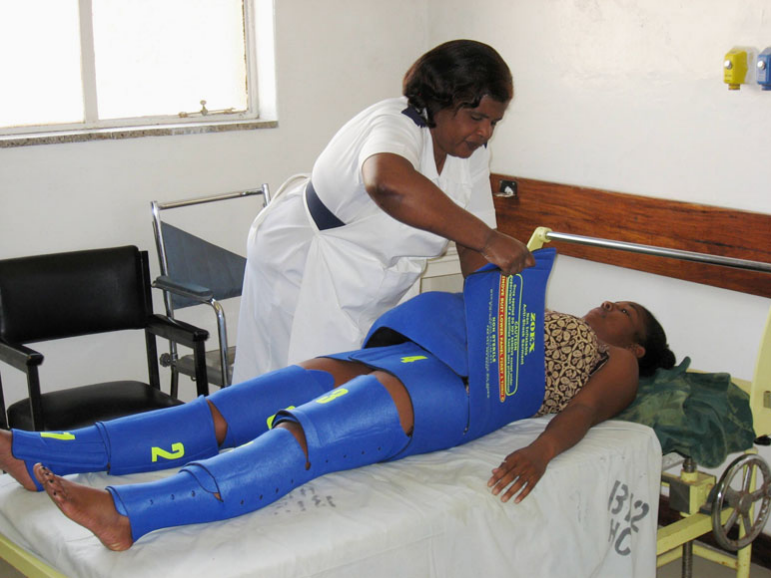
**The NASG being applied**.

Results from the use of the Non-pneumatic Anti-Shock Garment (NASG) (Zoex Corporation, Ashland, OR) to stabilize women with obstetric hemorrhage have been published in a case series, a pilot study, and two small comparative studies [3-7]. The NASG is a low-technology, first-aid device that delivers circumferential counter pressure to the lower body, legs, pelvis, and abdomen (Figure 1). Made of neoprene and Velcro™, with a foam compression ball that is placed over the abdomen, the NASG can be applied by anyone who has received training. Unlike the Pneumatic Anti-Shock Garment (PASG or medical anti-shock trousers MAST), previously used in the U.S. for pre-hospital trauma first-aid, there are no manometers, stop cocks or inflation devices; pressures applied by the NASG do not exceed 70 mmHg, thus avoiding potential ischemia or compartment syndrome. The mechanisms of action include applying circumferential counter pressure which decreases the container size; blood flow is decreased in the compressed area (abdomen, pelvis, lower extremities) while blood flow to the uncompressed area (core organs) is enhanced. Further, compression decreases the radius of the blood vessels in the abdomen and pelvis, including the splanchnic plexus, which decreases blood flow. More detailed information about the NASG has been published elsewhere [5-9].

We conducted non-randomized pre-intervention/intervention studies in four tertiary care facilities in Nigeria from March 2004 - December 2007 and in two tertiary facilities in Egypt from June 2006 - May 2008. The following analysis combines data from these facilities, which had equivalent pre-intervention and NASG data collection periods. The objective of this analysis is to examine whether adding the NASG to the standard protocol for managing hypovolemic shock secondary to obstetric hemorrhage of any etiology would improve maternal outcomes at the tertiary care level. While we have previously published the outcomes of interim analyses of a small (n = 169) study at one of the Nigerian facilities [7] and a larger one in Egypt (n = 990) [5], being able to combine the data from the two studies that were methodologically similar (using the same clinical diagnoses, protocol, and outcomes) allows us to be able to conduct more rigorous, robust regression analyses on a rare outcome: maternal mortality.

## Methods

The studies were approved by Institutional Review Boards at the University of California, San Francisco (UCSF), the National Reproductive Health Research Committee of the Nigerian Federal Ministry of Health, the El Galaa Maternity Teaching Hospital and Assiut University Women's Health Center.

The four sites selected from Nigeria were tertiary level teaching facilities, with 1,250-10,000 deliveries annually. The eight other facilities that were included in the project in Nigeria were implementation/intervention sites only and did not have a pre-intervention phase; therefore their data were not used in this analysis. The sites in Egypt comprised two tertiary teaching facilities, which had combined 31,990 deliveries in the pre-intervention and 31,176 deliveries in the NASG phase.

The methods of the studies have been described in more detail elsewhere [5,7]. Briefly, in both countries, non-randomized intervention studies with a pre-intervention phase for controls were conducted. Women with hypovolemic shock secondary to obstetric hemorrhage from any etiology were eligible for enrollment if they had an estimated blood loss of ≥750 mL and one or more clinical signs of hypovolemic shock (systolic blood pressure [SBP] <100 mmHg and/or pulse >100 beats per minute [BPM]). Women were eligible regardless if they began to hemorrhage outside the facility and were transferred in, or began to hemorrhage in the facility. All etiologies of obstetric or pregnancy-related hemorrhage were included: complications of abortion, ectopic pregnancy, trophoblastic disease of pregnancy, problems of placentation, ruptured uterus, abruption, uterine atony, and lacerations. Women with ante-partum hemorrhage >24 weeks with a living fetus (based on presence of fetal heart tones) were excluded.

During the pre-intervention phase in all facilities, women were managed with a standardized, evidence-based hemorrhage and shock protocol [5]; the intervention phase included the NASG into this protocol. The standardized protocol for both phases included: administration of oxygen, IV crystalloid fluids (>1500 mL in the first hour), and establishing the etiology of the hemorrhage. If the hemorrhage was due to uterine atony, uterotonics were administered, including: oxytocin, methergine, and misoprostol, and uterine massage or bimanual compression was performed. Depending on the source of the bleeding, the protocol included repair of lacerations, vaginal procedures such as manual vacuum aspiration or curettage to remove retained products and manual removal of retained placenta. Exploratory laparotomy was conducted and procedures performed, such as a salpingectomy for a ruptured ectopic, repair of a ruptured uterus, or an emergency hysterectomy. The protocol included laboratory investigations, such as complete blood count, creatinine, type and cross matching, and tests to rule out coagulopathies. Urine output was measured using either a Foley to a calibrated drainage bag or a straight catheter to a graduated collection bottle. Finally, the protocol called for a blood transfusion for all women with signs of shock.

Blood loss after study entry in both phases was measured using a closed-end, calibrated, plastic blood collection drape (BRASSS-V Fixable Drape™ Madurai, India). If the woman required a vaginal procedure, the NASG was left completely in place. If she required a laparotomy, the abdominal and pelvic segments were opened immediately prior to making the incision, and then replaced when the surgery was completed. Staff in the facilities were trained in the standardized protocol, blood collection and measurement, NASG use, and completion of data collection forms.

Definitions: For the purpose of the study, mortality refers to the death of a woman who was pregnant, post-partum or post abortion within the period of time of hospitalization for hemorrhage. Following the definition by Mantel et al.[10] severe maternal morbidities were defined as organ system dysfunctions related to severe obstetric hemorrhage: acute respiratory distress syndrome (impairment of respiratory function needing ventilation, oxygen supplementation or decreased physical activity level as compared to pre-pregnancy), cerebral impairment (seizures, unconsciousness, or cognitive/motor loss), renal failure (creatinine >1.5 mg/dL or increased >1.0 mg/dL above baseline, oliguria; <120 mL output in 4-hour intervals) and heart failure (impairment of cardiac function according to New York Heart Disease Classification [11]). The severity of the woman's condition at study admission was calculated by her mean arterial pressure (MAP); those with MAP <60 were in more severe condition. The formula for MAP is [2*diastolic blood pressure + systolic blood pressure]/3 [12].

Treatment variables included adherence to the clinical protocol of administering >1500 mL crystalloid fluids in the first hour after study admission, administration of blood transfusions, and administration of uterotonics for uterine atony. Outcomes of the study were mortality, severe end-organ dysfunction morbidity, measured blood loss, and emergency hysterectomy for uterine atony cases.

Paper and pen data collection forms were completed by trained clinician/data collectors at the time of treatment or shortly after. Data supervisors in each country reviewed the data forms for completeness and accuracy. Data forms were sent to the University of California, San Francisco (UCSF), entered into a Microsoft Access database (Microsoft, Redmond, WA, USA), checked for errors and inconsistencies and analyzed. In Egypt, the paper forms were sent electronically to UCSF through a data fax system (Clinical DataFax Systems Inc., Ontario, Canada).

Data Analysis: The analysis used combined data from the four largest Nigerian tertiary facilities (n = 452) and from two facilities in Egypt (n = 990); the total sample for analysis was 1442. The demographic characteristics, condition on study entry and treatment received for women in the two study phases were compared using two-sided t-tests of differences in the means of continuous variables (assuming unequal variances in the two study phase populations); the Wilcoxon rank sum test was used for non-normally distributed continuous variables (normality was tested using *qnorm *and *sktest *in Stata.), and chi-square tests of independence were used for dichotomous variables (with Fisher's exact tests used where required).

Relative risks (RR) with 95% confidence intervals were computed for the primary outcomes, mortality and severe maternal morbidity, and for the secondary outcome of emergency hysterectomy (for cases of primary or secondary diagnosis of uterine atony). From the RRs, we calculated the number needed to treat for benefit (NNTb) to prevent each of these outcomes, including an NNTb to prevent a case of *either *mortality or morbidity. An additional secondary outcome, the volume of measured blood loss in the drape (mL), was compared across study phases with the Wilcoxon rank sum test. To estimate the independent effect of the NASG intervention on mortality and severe maternal morbidity while controlling for other characteristics, we fitted a multiple logistic regression model for each of the two outcomes.

The independent variables included in the two models, in addition to study phase, were selected on the basis of their significant associations with the outcomes in bivariate analyses. These were severity of shock (MAP <60); parity (0-4 vs. 5 or more live births); primary definitive diagnosis of uterine atony vs. another diagnosis; and whether the woman began bleeding outside the facility or not (for the morbidity model only, as this variable was not significantly associated with mortality in bivariate analysis). In addition, facility was included as a control variable in order to hold constant the effect (on the outcomes) of any unmeasured systematic differences in the characteristics of the six clinical populations or in the quality of care provided in the six settings. We anticipated violations of the independent and identically distributed (I.I.D.) assumption in the data because of clustering at the facility level, which would cause standard errors in the regression models to be biased; therefore, we used robust standard errors (using *vce (robust) *post estimation command for logistic regression in Stata) to address this problem. The confidence level for all tests was set to 95%. Data were analyzed using Stata/SE (version 10).

Written informed consent was obtained for publication of the figure.

## Results

There were 1,442 women with hypovolemic shock entered into the study, 607 in the pre-intervention phase and 835 in the NASG phase. There were no significant differences in demographic characteristics (Table 1). There were a variety of etiologies, with significantly more ectopic pregnancy, ruptured uterus and placenta previa during the pre-intervention phase and more uterine atony, complications of abortion and lacerations during the NASG phase. During the pre-intervention phase significantly more women entered the study who had started bleeding at home/other facility vs. began bleeding in the hospital (p < 0.001). However, women in the NASG phase were in worse condition on study entry, with 38.5% (n = 321) with MAP <60 compared to 29.9% (n = 181) in the pre-intervention phase. Treatment variables (Table 2) show significantly fewer women in the NASG phase receiving either >1500 mL crystalloid fluids or a blood transfusion in the first hour (p < 0.001). However, by the end of the second hour after study admission, n = 531 (87.5%) in the pre-intervention phase and n = 723 (86.6%) had received the protocol (p = 0.62). The median time to initiation of the first blood transfusion, 35 minutes pre-intervention compared to 33 minutes NASG phase, was not significantly different.

**Table 1:** Demographics, diagnoses, and condition on entry to study (N = 1442)

	Pre-intervention N = 607	NASG N = 835	*p *value
**Study sites**			
Egypt (2 referral hospitals)	432	558	--
Nigeria (4 referral hospitals)	175	277	--

**Demographic characteristics**			
Age: Mean years of age (SD)	29.0 (6.4)	29.3 (6.2)	0.27
Median age (IQR)	28 (25-33)	29 (25-35)	--
Parity: Mean live births (SD)	3.2 (2.8)	3.3 (2.8)	--
Median parity (IQR)	2 (1-5)	3 (1-5)	0.18
Pregnancy duration: Mean weeks (SD)*	37.0 (4.1)	37.4 (3.6)	--
Median weeks (IQR)	38 (36-40)	38 (36-40)	0.18

**Primary definitive diagnosis**			
Uterine atony	190 (33.0)	319 (38.2)	0.007
Ectopic pregnancy	95 (15.7)	85 (10.2)	0.002
Complications of abortion	45 (7.4)	93 (11.1)	0.02
Abruption of placenta	79 (13.0)	98 (11.7)	0.47
Vaginal, cervical or genital lacerations	25 (4.1)	65 (7.8)	0.004
Retained placenta or tissue	71 (11.7)	83 (9.9)	0.29
Ruptured uterus	46 (7.6)	32 (3.8)	0.002
Placenta previa	40 (6.6)	31 (3.7)	0.01
Placenta accrete	6 (1.0)	9 (1.1)	1.000√
Molar pregnancy	7 (1.2)	11 (1.3)	1.000√

**Condition on study entry**			
Where hemorrhage began			<0.001
Transferred in bleeding	382 (72.9)	333 (56.4)	
Began bleeding in hospital	142 (27.1)	258 (43.6)	
Estimated revealed blood loss at study entry^+^			
Mean mL (SD)	1210.0 (507.7)	1327.5 (480.7)	--
Median mL (IQR)	1000 (1000-1500)	1200 (1000-1500)	<.0001
Women with MAP < 60 or non-palpable BP^**‡**^	181 (29.9)	321 (38.5)	0.001

NASG = Non-pneumatic Anti-Shock Garment. Data are n (column %), mean (SD) or median (IQR). The denominator is the entire population, unless otherwise noted. Tests of significance of differences by study phase were chi-square for categorical variables, t-tests (assuming unequal variances) for normally-distributed continuous variables and Wilcoxon rank-sum tests for non-normal distributions.

* Excludes abortion, ectopic and molar pregnancies.

√ Fisher's Exact test used.

^+ ^Data missing for 250 patients.

^**‡ **^MAP < 60 category includes those with non-palpable blood pressure (BP). Data missing for 2 patients.

**Table 2:** Treatments for shock and hemorrhage administered during two study phases (N = 1442)

Treatment	Pre-intervention N = 607	NASG N = 835	*p *value
Any uterotonics administered*	217 (96.9)	345 (97.7)	0.60^+^

≥1500 mL IV fluids within 1st hour^‡^	491 (81.2)	548 (65.6)	<0.001

≥1500 mL IV fluids within 2nd hour	531 (87.5)	723 (86.6)	0.62

Blood transfusion within 1^st ^hour	438 (72.2)	509 (61.0)	<0.001

Blood transfusion anytime after study admission	566 (93.3)	785 (94.0)	0.56

Minutes from study admission to 1^st ^blood transfusion ^§^			
Mean (SD)	119.1 (326.7)	117.5 (281.5)	--
Median (IQR)	35 (29-59)	33 (28-110)	0.48

NASG = non-pneumatic anti-shock garment. Data are n (column%). The denominator is the entire population, unless otherwise noted.

*Of women with uterine atony as primary or secondary diagnosis. Data are for 578 cases.

^+^Fisher's Exact test used.

^‡ ^The protocol was for 1500 mL to be administered in the first hour of resuscitation, however, in some cases only 1000 mL were administered in the first hour, while the remaining 500 mL were administered in the second hour. By the end of the second hour after study admission, n = 531 (87.5%) in the pre-intervention phase and n = 723 (86.6%) had received the protocol.

^§ ^Data for 104 patients missing (39 pre-intervention and 65 in intervention phase).

Outcomes were better for the women in the NASG phase (Table 3). Measured mean and median volumes of blood loss after study entry were lower, medians were 400 mL in the pre-intervention phase vs. 200 mL NASG phase (p < 0.001). Emergency hysterectomies for intractable uterine atony were reduced by 56%, from 8.9% (n = 20) pre-intervention to 4.0% (n = 14) in the NASG phase (RR 0.44, 95% CI 0.23-0.86). Severe morbidities were decreased by 81%, with 3.7% (n = 21) experiencing end-organ dysfunction in the pre-intervention phase vs. 0.7% (n = 6) in the NASG phase (RR 0.20, 95% CI 0.08-0.50) and mortalities were reduced by 44%, 6.3% (n = 38) died in the pre-intervention while only 3.5% (n = 29) died during the NASG phase (RR 0.56, 95% CI 0.35-0.89). The NNTb to avoid an adverse outcome was also computed from the RR statistics [13], for the following outcomes: the NNTb to prevent maternal mortality was 36, (95% CI 20-202), the NNTb to prevent one severe morbidity was 34 (95% CI 22-78). For women with a diagnosis of uterine atony, the NNTb to prevent one emergency hysterectomy was 20 (95% CI 11-138). To prevent one extreme adverse outcome, combining mortality and severe maternal morbidity, the NNTb was 18 (95% CI 12-36). (Data not shown)

**Table 3:** Outcomes between standard hemorrhage and shock management (pre-intervention) and standard management plus NASG (intervention) (N = 1442)

Outcome	Pre-intervention (N = 607)	**NASG (N = 835**)	Relative Risk (95%CI)	*P *value	NNTb
Measured vaginal blood loss in drape:					
Mean mL (SD)*	443.5 (346.1)	240.0 (199.4)		--	
Median mL (IQR)	400 (250-500)	200 (150-250)		<0.0001	

Emergency hysterectomy^+^	20 (8.9)	14 (4.0)	0.44(0.23-0.86)	--	20 (11-138)

Morbidity^**‡**^	21 (3.7)	6 (0.7)	0.20 (0.08-0.50)	--	34 (22-78)

Mortality	38 (6.3)	29 (3.5)	0.56 (0.35-0.89)	--	36 (20-202)

NASG = Non-pneumatic Anti-Shock Garment. Data are n (%) or mean (SD). The denominator is the entire population, unless otherwise noted.

* For cases in which the calibrated blood collection drape was used and there were data for blood loss. Wilcoxon rank-sum test used to compare distributions by study phase. Data are for 1005 cases.

^**+ **^Data on emergency hysterectomy are only for women with primary or secondary diagnosis of uterine atony (n = 578).

^**‡ **^Includes renal failure, acute respiratory distress syndrome, heart failure, cerebral impairment (seizures, un-consciousness, motor/cognitive loss) lasting more than 24 hours after resuscitation from shock. Denominator is the number of women who survived (n = 1375).

The results of the multiple logistic regression analysis are shown in Table 4, with the left column of the table denoting findings for the model with mortality as the dependent variable, and the right column showing findings for the morbidity model. As shown, women with a MAP <60 mmHg had over eight times the odds of mortality (aOR 8.42, 95% CI 3.13-22.66) relative to those with MAP ≥60 mmHg. No other control variables (parity, primary diagnosis or facility) were significantly associated with mortality, but the NASG intervention was associated with 55% lower odds of mortality (aOR 0.45, 95% CI 0.27-0.77). In the model of factors associated with severe maternal morbidity, women with a MAP <60 mmHg had almost five times the odds of morbidity (aOR 4.83, 1.80-12.94) relative to those with MAP ≥60 mmHg. Those with a parity of 5 or more had 2.4 times the odds of morbidity (aOR 2.43, 1.06-5.58), but both where bleeding began and facility were not associated with the outcome. The NASG intervention was significantly associated with 80% lower odds of morbidity (aOR 0.20, 95% CI 0.07-0.56).

**Table 4:** Multiple logistic regression models of factors predictive of mortality and morbidity

Factor	Dependent variable: mortality				Dependent variable: morbidity			
	
	aOR	*p*	95% CI		aOR	*p*	95% CI	
**Severity of shock**								
MAP < 60 (or non-palpable BP)	8.42	<0.001	3.13	22.66	4.83	0.002	1.80	12.94
*MAP 60 or higher*	*1*				*1*			

**Parity**								
5 or more live births	1.33	0.35	0.73	2.42	2.43	0.04	1.06	5.58
*0-4 live births*	*1*				*1*			

**Primary Diagnosis**								
Uterine atony	1.44	0.19	0.83	2.49	2.68	0.07	0.93	7.76
*Other condition*	*1*				*1*			

Where bleeding began*								
Transferred in bleeding	--	--	--	--	1.82	0.51	0.30	10.93
*Began bleeding at RH*	--	--	--	--	*1*			

**Study Phase**								
NASG	0.45	0.004	0.27	0.77	0.20	0.002	0.07	0.56
*Pre-intervention*	*1*				*1*			

NASG = Non-pneumatic Anti-Shock Garment. aOR = adjusted odds ratio. Reference groups for categorical variables shown in italics. Hospital facility included as control variable in both models, but not shown in Table 4. The number of observations in Table 4 is less than 1442 because of missing data; n = 1038 for the morbidity model, and n = 1442 for the mortality model. Robust standard errors used to adjust for clustering at the facility level.

* Where bleeding began was not a significant predictor of mortality, but it was associated with morbidity, in bivariate analysis. Therefore it is included in the multiple logistic regression model of factors predictive of morbidity only.

Because the odds of morbidity and of mortality were so strikingly high for women with MAP <60, independent of the study phase, we also conducted a stratified analysis by severity of condition (MAP <60 vs. MAP ≥60) for each of the two outcomes, using the same model specification from Table 4. An ameliorative effect of the intervention for reduced morbidity was seen in both women with MAP <60 (aOR 0.20, 95% CI 0.05-0.80,) and MAP ≥60 (aOR 0.18, 95% CI 0.04-0.90). For the stratified mortality model, the NASG intervention was significantly associated with a reduced odds of death in women with MAP <60 (aOR 0.46, 95% CI 0.26-0.80), but not in women with MAP ≥60 (aOR 0.68, 95% CI 0.14-3.22). (Data not shown)

## Discussion

In these six tertiary care facilities in Nigeria and Egypt, women with hypovolemic shock secondary to obstetric hemorrhage treated with standard protocol plus the NASG had improved outcomes, despite being in worse condition on study entry than those in the pre-intervention phase.

The results of multiple logistic regression modeling of the independent associations of the NASG with mortality and morbidity respectively, suggest that the NASG intervention shows promise for reducing the incidence of extreme adverse outcomes in women with hypovolemic shock secondary to obstetric hemorrhage. The concern motivating our stratified analysis of factors associated with morbidity and mortality by MAP grouping was to determine if the intervention was only effective in women in less severe shock; the findings underscored the NASG's association with reduced morbidity and mortality even in women with MAP <60, and supports the intervention's potential efficacy. The lower rates for mortality and severe morbidity among women with MAP ≥60 may account for the lack of significance for that variable.

An interesting finding among this large sample of women with severe hemorrhage is the relatively low rates of uterine atony. While the contribution of uterine atony to complications of pregnancy and maternal mortality have been described as anywhere from 40-70% [14,15], in this study where only women suffering severe hemorrhage and shock were entered, uterine atony accounted for less than 40% of the sample. This study began before the practice of the Active Management of Third Stage Labor (AMTSL) was well established in either country, so it is possible that as AMTSL becomes more widely practiced, uterine atony will be seen as contributing even less to maternal mortality from obstetric hemorrhage. Nearly all the women with uterine atony in both phases received uterotonics for treatment. In terms of outcomes, significantly fewer women in the NASG phase underwent emergency hysterectomy for uterine atony, which may be attributable to the lack of panic among providers who were caring for women with decreased bleeding and stable vital signs. A recently published sub-analysis of 854 women from the combined Egypt and Nigeria dataset, all of whom had a PPH diagnosis, revealed similar findings to the obstetric hemorrhage outcomes shown here, with a mortality decrease from 9% pre-intervention to 3.1% in the NASG phase (RR 0.35, 95% CI 0.19-0.62) [16].

It is also interesting to note the worse condition of the women on study entry into the NASG phase. We believe that this was due to the clinician/data collectors hesitancy to put on the NASG until the woman was severely compromised, given that the NASG was a new technology, and the clinicians had not yet incorporated it into their emergency response repertoire. They may have delayed putting it on until it was clear that the women really needed it. While an alternative explanation could be that the women became more ill during the time the NASG was applied, it is unlikely, given that the NASG can be applied in less than 3 minutes. Another finding, the lower rate of women receiving the correct volume of IV fluids for shock resuscitation, may have been due to a developed complacency after placing women in the NASG. Once the woman, regardless of her state of shock, is placed in the NASG, the response is seen almost immediately: the pulse slows, the BP rises, the woman regains consciousness, and the bleeding diminishes. It may be that clinicians seeing this response to placement of the NASG may have been less anxious. If this is the case, it means that training must be modified to stress that all women with hypovolemic shock need the same immediate standard resuscitation, and that placement of the NASG is supportive of, but does not replace the need for IV fluids, blood, and other hemorrhage and shock management. While NASG use decreases mortality, women still die with the NASG, particularly if there are major delays in supplying blood [17].

There are limitations inherent in the pre-post study design. The effect of time and experience managing women in hypovolemic shock could have improved over time, as the intervention succeeded the pre-intervention. Selection bias is always a risk in a non-randomized trial, where not every patient in a facility is enrolled. It may have been that there were patients who were not enrolled despite meeting entry criteria. Given the large number of patients in each facility and the lack of staffing, some patients' data may not have been entered. Another limitation to this study was that there was an imbalance between patients based on both etiology of hemorrhage and on where patients began bleeding. More patients in the NASG phase had uterine atony, while more patients in the pre-intervention phase began bleeding outside the facility. In bivariate analysis, where bleeding began was not significantly associated with mortality, but it was associated with morbidity; however, it did not remain significantly associated with morbidity in the multiple logistic regression model. Uterine atony diagnosis was associated with both adverse outcomes in bivariate analyses, but the association did not remain statistically significant in either of the multiple logistic regression models.

There are limitations to the applicability of these findings to the real world of maternal mortality and morbidity due to obstetric hemorrhage in low-resource settings. With the majority of the world's women delivering outside of tertiary facilities and without skilled attendants, there may be greater implications for public health in a study conducted outside the tertiary care level. A randomized trial with women entered into the study at peripheral levels is now being conducted in Africa (http://www.clinicaltrials.gov: NCT00488462).

## Conclusions

Obstetric hemorrhage is the leading cause of maternal mortality globally, particularly in low-resource settings where long delays contribute to adverse outcomes that would not occur in higher resource settings. The use of the NASG, a low-technology, circumferential counter pressure first-aid device, appears to mitigate the effect of the delays. More rigorous research is warranted to better understand the potential of this device.

## Competing interests

The authors declare that they have no competing interests.

## Authors' contributions

SM conceived of the study, obtained funding, participated in study design and coordination, acquired data and drafted the manuscript. MF participated in the conception of the study, acquired data, administered and managed the study, and helped draft the manuscript. OAO acquired data and administered and managed the study. CC performed statistical analyses. MMY participated in the conception of the study, acquired data, administered and managed the study, and helped draft the manuscript. IOM acquired data and administered and managed the study. HG acquired data and administered and managed the study. DN acquired data and administered and managed the study. EB administered and managed the study. TA acquired data and administered and managed the study. JT performed statistical analysis. CM helped draft the manuscript and performed statistical analyses. HM managed data cleaning and performed statistical analysis. AIM acquired data and administered and managed the study. All authors read and approved the final manuscript.

## Pre-publication history

The pre-publication history for this paper can be accessed here:

http://www.biomedcentral.com/1471-2393/10/64/prepub
